# The equine species as Trojan horse for Borna Disease Virus-1?

**DOI:** 10.1080/01652176.2019.1551172

**Published:** 2019-02-18

**Authors:** J.H. van der Kolk

**Affiliations:** Editor-in-Chief, Swiss Institute for Equine Medicine (ISME), Vetsuisse Faculty, University of Bern, Bern, Switzerland

Dear reader,

The recent report on a veterinarian bitten by a horse seropositive to Borna Disease Virus-1 (BoDV-1) in the Netherlands (Sloet van Oldruitenborgh-Oosterbaan et al. 2018) draws attention to the zoonotic potential of this virus.

BoDV-1, the cause of most cases of mammalian Borna disease, is a RNA virus that replicates within the nucleus of target cells. It causes severe, often lethal, encephalitis in susceptible species (Tizard et al. 2016).

Recently, three breeders of variegated squirrels (*Sciurus variegatoides*) in Germany had encephalitis with similar clinical signs and died 2–4 months after onset of the clinical symptoms associated with variegated squirrel 1 Bornavirus (VSBV-1) (Hoffmann et al. 2015). In addition, in psittacine birds with proventricular dilatation disease (PDD) a severe lymphoplasmacytic ganglioneuritis of the gastrointestinal tract is frequently accompanied by encephalomyelitis associated with avian Bornavirus (ABV) (Staeheli et al. 2010). These recent events have revived interest in this remarkable family of viruses (Tizard et al. 2016).

BoDV is an enveloped, nonsegmented negative-stranded neurotropic RNA virus classified in the virus order Mononegavirales similar to rabies virus. Borna disease was first described as a meningoencephalitis of horses. The name Borna reflects outbreaks in the vicinity of the town Borna, in Saxony, Germany, wherein large numbers of animals died in the late 1800s (Lipkin et al. 2011). Furthermore, Borna disease has also been reported in sheep, cattle, llamas, cats, dogs and ostriches. Because an even larger variety of species has been experimentally infected, including rabbits, birds and primates, the potential host range includes all warm-blooded animals. Natural BoDV infection has been reported primarily in Europe (Lipkin and Briese 2007). Of note, signs of BoDV infection, including antibodies, antigen, RNA and/or virus itself, have been reported from animals in many continents. The highest clinical incidence in animals and the verified classical Borna disease cases, however, are restricted to central Europe (Staeheli et al. 2000; Pawaiya et al. 2010; Kinnunen et al. 2013).

Shrews are regarded as reservoir hosts of BoDV (Hilbe et al. 2006). The incidence of Borna disease in horses and sheep peaks in March to June (Kinnunen et al. 2013). An olfactory route for transmission has been proposed because intranasal infection is efficient and the olfactory bulbs of naturally infected horses show inflammation and edema early in the course of disease (Ludwig et al. 1988). In man, herpes simplex virus type 1 (HSV-1), human herpesvirus 6 (HHV-6), Borna disease virus, rabies virus and influenza A virus have also been shown to take the olfactory route for neuroinvasion (Mori 2015).

**Figure 1.**
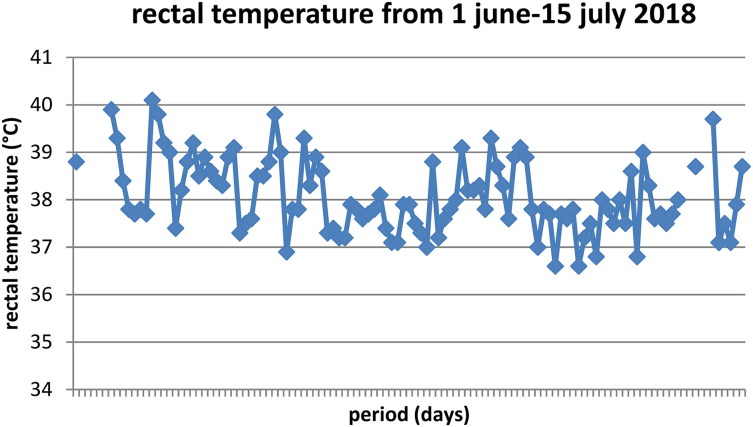
Daily rectal temperature over time in a 7-year-old warmblood gelding (1:160 seropositive to BoDV-1) prior to euthanasia due to continuous neurologic signs like ataxia. The fever persisted despite treatment with antibiotics and NSAIDs (reprinted with permission from Van der Straaten et al. 2018).

After an incubation period lasting a few weeks to several months, BoDV infection can cause locomotor and sensory dysfunction followed by paralysis and death (Richt et al. 2000). The neurological course in horses usually begins with excitability or depression and ends with severe excitability, aggressiveness or lethargy, and circling, paresis, paralysis, somnolence, stupor and coma (Kinnunen et al. 2013). Fever (see Figure 1), anorexia and ataxia are characteristically described (Katz et al. 1998; Pawaiya et al. 2010; Kinnunen et al. 2013). Blindness due to loss of photoreceptors (Dietzel et al. 2007) and colic have also been reported (Kinnunen et al. 2013). It should be realized that the infection with BoDV in horses can exist without associated clinical symptoms. Furthermore, the majority of natural BoDV infections occur unnoticed as approximately 43% of the infected horses were clinically ill (Dieckhöfer 2008). Ponies infected experimentally with BoDV through intracerebral inoculation seroconvert one-month post inoculation (Katz et al. 1998). Of note, it has been stated that infected animals produce BoDV-specific antibodies only after virus replication (Richt and Rott 2001). The ensuing period of neurologic dysfunction ranged from 3 to 16 days following intracerebral injection and two ponies died after rapid onset of these signs 28–30 days post inoculation (Katz et al. 1998). Well known are the pathognomonic Joest-Degen inclusion bodies in the post mortem brains (Dietzel et al. 2007).

Rabies virus causes an acute lethal encephalomyelitis with only minor inflammatory reaction, whereas infection with BoDV results in persistent CNS infection characterized by massive infiltration of inflammatory cells (Fu et al. 1993). Furthermore, rabies virus infects only neurons, whereas BoDV also infects glial cells (Gosztonyi et al. 1993). The nucleocytoplasmic transport of BoDV macromolecules is an essential component of the life cycle of BoDV (De La Torre 2002). While in the later phases of replication complete rabies virions are regularly assembled, BoDV propagates within the central nervous system in an incomplete form, so that it remains morphologically imperceptible. Thus, BoDV may appear in a complete, enveloped form only when exiting the host organism. It remains unresolved, why BoDV readily infects non-neuronal central nervous system cells, while rabies virus remains restricted to neuronal elements (Gosztonyi et al. 1993). BoDV-infected equine hippocampi were characterized by lower levels of d-myo-inositol-1-phosphate, glutamate, phosphoethanolamine, heptadecanoic acid and linoleic acid in combination with a higher level of ammonia, which differential metabolites are primarily involved in glutamate and lipid metabolism (Zhang et al. 2014). Neurons infected by BoDV may be destroyed by T-cell-mediated cytotoxicity and they may die either as a result of excessive inflammatory cytokine release from microglia or as a result of a ‘glutaminergic storm’ due to a failure of infected astrocytes to regulate brain glutamate levels (Tizard et al. 2016).

It has been suggested that the tight confinement of the virus to the CNS compartment, which is most likely the result of a strong antiviral cellular immune response, argues against the possibility of viral shedding by infected horses (Weissenböck et al. 2017). In contrast, possible vertical transmission of BoDV in a Japanese horse has been implicated as the brain of the pregnant mare and the histologically normal brain of the fetus were both positive for BoDV RNA (Hagiwara et al. 2000). Furthermore, the presence of BoDV-specific RNA was traced in conjunctival fluid, nasal secretions and saliva of horses which were seropositive but did not have any history of clinical Borna disease (Richt et al. 1993, Lebelt and Hagenau 1996). In addition, BoDV-specific RNA was detected in kidney and bladder in naturally infected animals with clinical disease (Lebelt and Hagenau 1996). However, viral infectivity or virus-specific antigen was not found in any of these secretions by conventional assays in cell culture and immunoblotting (Richt et al. 1993). Last but not least, the seroprevalences (2.6–14.8%) of BoDV were significantly higher in the blood donors from four regions of Hokkaido island, Japan where most horse farms are concentrated, compared with only 1% in the blood donors from Sapporo, the largest city in Hokkaido indicating that BoDV may be horizontally transmitted, at least in part, from infected horses to humans (Takahashi et al. 1997).

The matter is further complicated by the fact that recent research showed that BoDV sequences are incorporated into the genome of humans and other mammals (Horie et al. 2010; Belyi et al. 2010) indicating that Bornaviruses previously infected primates more than 40 million years ago (Lipkin et al. 2011).

As carefully verified human BoDV infections have occurred rarely (De La Torre et al. 1996), but because transmission between man and vertebrate animals has not been demonstrated clearly yet, BoVD remains a possible, not verified, zoonosis (Kinnunen et al. 2013).

J.H van der Kolk
Editor-in-Chief
*Swiss Institute for Equine Medicine (ISME), Vetsuisse Faculty, University of Bern, Bern, Switzerland*
johannes.vanderkolk@vetsuisse.unibe.ch

